# Book Reviews

**Published:** 1822-04

**Authors:** 


### CRITICAL ANALYSIS

OF

### ENGLISH AND FOREIGN LITERATURE

RELATIVE TO THE VARIOUS BRANCHES OF

### Medical Science.

Quæ laudanda forent, et quæ culpanda, vicissim

Illa, prius, cretâ; mox hæc, carbone, *notamus*.—PERSIUS.

### DIVISION I.

### ENGLISH.

ART. I.—*Essays on Surgery and Midwifery; with Practical Observations and Select Cases.* By JAMES BARLOW, Surgeon. With Plates. 8vo. pp. 417. Baldwin and Co. London, 1822.

WE are always glad to meet with a respectable country practitioner, who can spare sufficient time to record the many judicious observations he may have had occasion to make in the course of his experience; and who can relate, in a simple and unassuming style, the facts he may have witnessed, without, at the same time, pretending to explain, or build a theory on them, for the edification of his readers.

Had Mr. BARLOW confined himself to a plain, unvarnished tale of the results of his practice, whether in pure surgery or obstetrical medicine, we doubt not but that his book would have been received with unalloyed gladness. As it is, we fear there will be many readers who will object to the dogmatic and elementary exposition of surgical or obstetrical principles, with which his facts are encumbered; while others will reject the facts themselves, merely because they are brought forward as the sequelæ of pedantic demonstrations.

We can conceive that a country practitioner, like HAY, who invented or improved surgical instruments,—simplified operations,—suggested new modes of treatment in rebel disorders,—may be listened to attentively, and be looked up to as an authority, whenever he embodies the fruit of his long experience in a series of rules, or in a book of elementary principles. Such a book will always be considered as a work of reference, in cases of doubt; and one in which professional information, whether of a general or a private character, will be sought for. But Mr. Barlow is placed in very different circumstances. The profession know only of his having related a case of Cæsarean operation, successfully performed by himself, which, at the time, gave rise to much sceptical discussion. Nor is it likely that, upon such slender claims to popularity, surgeons, whether young or old, will take up his present essays with an expectation of deriving, from a perusal of them, that generic kind of knowledge which is to be found in the more classical and richly-laden pages of a metropolitan surgeon, whose opportunities for the improvement of his profession and the generalization of facts, are only equalled by the eagerness with which he avails himself of them for the interest of others.

Mr. Barlow, therefore, we apprehend, did not judge rightly in suffering himself to be induced to enter into long elementary discussions, or anatomical descriptions, (which, at most, are but superficial,) respecting points of surgery and obstetricism; the history, nature, and treatment of which are already familiar to the profession. He ought to have confined himself to a plain statement of those facts he had himself observed in his practice, and he would have then presented us with, we have no doubt, much valuable and practical information.

But, even fashioned as it is at present, the volume before us embraces much that is worthy of perusal; and we, therefore, proceed to give an analysis of its contents. It is amusing to see how early propensities will, sometimes, induce us to invert the natural order of things. Whenever Mr. Barlow has occasion to associate the two words MEDICINE and SURGERY, he has invariably assigned the first place to the latter. He, moreover, talks of the *philosophy* of surgery! For all these singularities of Mr. Barlow, there is, however, a very ample excuse, alledged by the author himself, who observes, that he has "frequently lamented that, when his professional career began, the same requisitions were not enforced, and, of course, the same advantages of scientific instruction were not generally professed; and the locality of his situation has also deprived him of these professional advantages." (p. vi. and vii.)

The requisitions here alluded to, Mr. Barlow has previously told us, are those enacted by late legislative arrangements; by which, we presume he means the Apothecary's Act, which renders it imperative, now-a-days, on any one desirous of entering the medical profession, to study medicine before he practises it; a law which did not obtain when Mr. Barlow began his career. This is perfectly ingenuous.

But, although education may have been deficient, zeal, industry, and attention, have not been wanting to make up for it. Mr. Barlow has been in the habit of committing to writing the result of his inquiries; and it is to this praise-worthy practice that we are indebted for the volume we have now brought before our readers.

"From a variety of papers he has selected such as have excited, on his part, the greatest degree of attention; and, though some of the essays were communicated by the author to the periodical journals, in the early part of his practice, yet time and experience have suggested those improvements and corrections, which have enabled him to enlarge considerably his original communications. He trusts that what he has published will be received with that liberality and candour which are the best ornaments of professional excellence. He has no wish to disarm the severity of criticism by acknowledgments or professions, because he makes no pretensions beyond such as are founded on a sincere desire to advance the interests of surgical science, and to facilitate the operations of surgical skill." (p. vii. and viii.)

The first part of Mr. Barlow's volume is dedicated to the consideration of some diseases of the urinary organs. This is preceded by some general observations, in which the study of the structure of the pelvis, and functions of its connected viscera, is strongly recommended. Some of the affections of the prostate are slightly alluded to; and, among the causes of this last class of diseases of the urinary apparatus, Mr. Barlow mentions "high living, constipation of the bowels, and excessive sexual intercourse."

We do not profess to understand, and, if we did, we could not acquiesce in, the explanation proposed by our author of the formation of calculi.

"When there exists in the constitution a predisposition to the formation of calculi, portions of gravel, which pass from the ureters, are retained in the cavity of this receptacle till a nucleus is formed; and this may be one way in which calculi are primarily produced, and eventually become increased to a size too large to pass through the urethra naturally. Thus one disease frequently tends to aggravate another, and, in this instance, a retention of urine at intervals occurs: hence the aid of the catheter is required as a means of affording temporary relief to the patient."

In cases of pain or irritation of the bladder, whether from the presence of calculi or some morbid affection of its coats, accompanied by an irresistible solicitation to urine, and a painful sensation in the glans penis or neck of the bladder, Mr. Barlow has used, with great success, an injection, consisting of a solution of opium, or an infusion of the leaves of belladonna, and mucilage of gum arabic, in tepid water, into the bladder. Sometimes the passing of a bougie, though there be no stricture in the urethra, is attended with evident advantage.

Amongst the various causes of retention of urine, one is mentioned by our author, proceeding from active inflammation of the prostate gland, extending up the urethra, the tender vessels of which become so surcharged with blood, that, on every gentle attempt to introduce the catheter, the eyes of the instrument get so completely blocked up before it enters the bladder, that no urine can pass through the tube. To obviate this sort of obstruction in the catheter, Mr. Barlow says that he usually plugs up the eyes of the instrument with wax, before its introduction, in the expectation that the latter will melt, or that it will be forced out by blowing into the catheter, and thus the urine made to flow without interruption. This is both new and ingenious. There are only two difficulties to its execution: the first, that wax will not melt at the temperature usually found in the urethra, especially as moisture is present; and the second, that where the urethra itself is stated to be so surcharged with blood that the eyes of the catheter are plugged up with them, when an attempt is made to introduce it—there will be no room for *blowing out* the said wax. Mr. Barlow, therefore, must give us a better explanation of his success in these cases.

Every surgeon prides himself in possessing a certain degree of dexterity with regard to the introduction of the catheter. Mr. Barlow is not deficient in this sort of feeling; and our readers will learn from himself how readily he can perform that operation, where other practitioners have failed.

"Retention, or a defect in the evacuation of the urine, is always a serious disease, and may be produced by a variety of causes; such as, mechanical or morbid obstructions of the urethra, prostate gland, or inflammation of the bladder; for, when this receptacle becomes extensively distended for a length of time, it loses its power of contraction, and, unless relieved by the catheter in due time, the urine becomes acrid, and inflammation and mortification are the consequences. This calamity is more frequently incidental to the advanced state of life, and its morbid effects are, in some degree, counterbalanced by the system being less susceptible of irritation and stimuli than at an earlier period. I have known many old men retain their urine, with comparative impunity, for the space of two of three days; and in one instance a gentleman, by whom I was consulted, was six days and nights without voiding any urine. During the whole of that period, the catheter and other means had been fruitlessly employed by two medical attendants, who considered the disease to be a suppression of the secretion of urine. In this case, instantly and without difficulty, I passed the catheter into the bladder, and evacuated more than three quarts of turbid offensive urine; and, on passing the finger up the rectum, I discovered an enlarged prostate gland, which induced me to leave a flexible catheter, with a small calf's-bladder affixed to it in this viscus, through which the urine was permitted to pass off *ad libitum.* The instrument was taken out and cleaned occasionally, at which times I could distinctly feel a calculus with its point; and, after the lapse of a few weeks, the organ resumed its natural functions. The inflammatory symptoms having subsided, I became anxious to ascertain the natural power of the bladder; for which purpose the catheter was left out, and in a few hours the gentleman was seized with excruciating pains about the neck of the bladder. On attempting to pass the instrument, I perceived the calculus had got into the urethra, which led me to pass the finger up the rectum, with which I pressed the calculus forwards, whilst the point of the catheter served to distend the passage. In this manner I extracted a calculus about the size of a horse-bean, and the instrument was replaced in the bladder as before. During this period the gentleman was allowed to walk about; and, though he has since experienced occasional attacks of retention of urine, the same mode of treatment being adopted, his life has been preserved, and he now enjoys a good state of health in the eighty-fifth year of his age. In addition to mechanical aid, these cases require a strict attention to the antiphlogistic course, according to the age and condition of the patient. If there be a full quick pulse, with flushings of the face, and much fever, general as well as local, bleeding will be proper; such as cupping from the pubis or perineum." (p. 20—22.)

The second and third point connected with the diseases of the urinary organs, respecting which Mr. Barlow has favoured us with the result of his experience, are the method of sounding for the stone, and a knowledge of the symptoms of its presence in the bladder.

"To acquire an accurate diagnosis of the presence of stone in the bladder," observes the author, "it is proper to question the patient respecting the primary symptoms: whether they originated in the kidneys, or in the bladder; whether small calculi, or sabulous depositions, have been observed in the urine; and whether there be any cause to suspect an hereditary diathesis.

"The symptoms usually accompanying stone in the bladder are gradually developed, and may be included in the following statement:

"A sense of weight and uneasiness felt about the neck of the bladder, and pressure at the rectum, particularly when the patient moves about.

"An irresistible propensity to a frequent expulsion of the urine, although the bladder may have been emptied not long before, especially when in an erect position, along with a smarting, burning, or pricking sensation at the extremity of the penis, which causes the patient to walk in a creeping straddling manner; with an almost incessant desire to go to stool. The urine, in the incipient stage, is almost always clear; but, as the disease advances, it becomes turbid, caused by repeated attacks of inflammation, which produce ulceration, and a secretion of coagulable lymph from the inner surface of the bladder. Eventually, pus is perceived to be blended with the urine, and streaks of blood, more or less as the surface of the calculus is rough or smooth; and, during the progress of the disease, the desire to pass urine becomes more and more frequent, till, at length, the bladder loses its usual power of distension, hectic fever ensues, and the patient sinks under the disease, unless the stone be removed.

"Great pain at the glans penis on ejecting the last drop of urine, connected at other times with tenesmus, more particularly in young persons; an intolerable itching irritation about the external orifice of the urethra, attended with frequent involuntary erections of the penis, which part the patient is induced to nip with his fingers, to relieve the paroxysms, while the prepuce becomes elongated, and sometimes nearly closed, are also among the symptoms of this disease.

"In some instances, the sufferer is free from pain for a certain time, and a considerable quantity of urine will be collected in the bladder, which, on evacuation *pleno rivo,* is suddenly stopped; but, on the patient varying the position of his body, the calculus recedes from the neck of the bladder, and the urine flows again freely as before.

"In the advanced stage of the disease, the urine deposits a sabulous and turbid sediment, with an offensive smell. On some occasions, the abdominal muscles become affected with spasm, and the patient is seized with fits of shivering, accompanied with a discharge of mucus along with the urine, which indicates inflammation of the coats of the bladder: under these circumstances the operation is inadmissible.

"Difficulty of retaining the urine, with dribbling resembling stillicidium urinæ, and, at intervals, urgent efforts to expel the contents of the bladder, caused either by a change in the situation of the stone, position of the body of the patient, or the coats of the bladder being no longer protected by the intervention of the urine. During these efforts the difficulty becomes greater, and the pain aggravated.

"Hæmorrhoids in the decline of life, and prolapsus ani in children, are frequent attendants on the disease, together with occasional retraction and atrophy of the testicles.

"When small calculi become lodged in the ducts of the prostate gland, there is usually some difficulty in voiding the urine, attended with pain about the neck of the bladder, and a sense of uneasiness at the glans penis, not very dissimilar to that produced by a stone in the cavity of the bladder; in which case, the surgeon should aid the researches of the sound, by passing the finger up the rectum, in order to acquire a more correct diagnostic of the nature of the case." (p. 32—34.)

But even the assemblage of all the above indications may unwarily mislead the surgeon, unless he should, at the same time, have recourse to the assistance of the sound, ere he decides on the case.

Mr. Barlow then proceeds to tell us what a sound is, and how it should be used; precisely as if the profession were but little informed on either of those subjects. He likewise proposes an improved instrument of that name, which he has reduced to about one-half of the usual thickness or diameter, on the supposition that its motion, in passing down the canal of the urethra, will thereby be greatly facilitated.

Mr. Barlow has also favoured us with details of his method of introducing the sound in the male subject, which, he observes, may be done when the patient is standing, sitting, or lying.

"Thus, placing myself before the patient when in the first position, so as to have full command of the instrument, which is to be adapted to the size of the urethra, and previously warmed and besmeared all over, except the handle, with oil, I take a gentle hold of the corona glandis between the thumb and two fore-fingers of the left hand, raising the penis a little upwards; and, with the handle held in the right hand, between the index and fore-finger, I then insinuate its point into the aperture of the urethra, with the convex part towards the patient, pressing it steadily onwards till it meets with some resistance *in perineo,* from the curvature of the passage that corresponds to the arch of the pubis. Keeping the point fixed in this position, as the centre of motion, and the penis at the same time raised on the instrument, I then, with the same hand, make a semi-circular turn upwards, over the left groin of the patient, which brings the hand parallel with the navel; and, at the same time, inclining the handle of the catheter forwards horizontally, by one continued and uninterrupted sweep of the hand, I depress the handle, by which its point will naturally, with a little force, ascend under the angle of the symphysis pubis, and enter the cavity of the bladder."

Mr. Barlow, as it were *en passant,* observes, that a want of a chemical agent to dissolve a stone in the living bladder, is a matter of regret; and that, until this *arcanum* be discovered, lithotomy will ever remain an indispensable expedient.

Having thus led his readers to the threshold of lithotomy, Mr. Barlow proceeds, in the ensuing chapters, to give an historical description of the different methods of performing that operation, with introductory remarks. The first described is the Celsian operation, or what was denominated the apparatus minor. The apparatus major, or *sectio mariana*, follows; and the high operation, or apparatus altus, is next detailed. After giving an account of Rousset's method of performing the high operation, Mr. Barlow enumerates the advantages and disadvantages of that operation, as more commonly performed, and seems rather inclined towards it.

On the lateral operation Mr. Barlow has entered very copiously, giving the history of its first introduction by Frère Jaques, and a minute description of the present mode of performing it, either with the knife or the gorget; to the former of which instruments our author appears to give the preference.

Respecting lithotomy on women, Mr. Barlow has only made a few common-place observations, which need not be analyzed in this place. In performing the operation on women, our author thinks that, in almost all cases, the *bistouri caché* appears to be preferable. When called upon to operate on men, Mr. Barlow has employed either the *bistouri caché* or a beaked lithotomy knife of his own invention; and both of them with a success that will surprise many hospital-surgeons. Mr. Barlow states that he has operated on upwards of sixty patients for the stone, out of which number only two have not survived the operation.

We shall pass over Mr. Barlow's cases, and his article on tumor of the nose, as they have appeared before in this Journal and other publications.

The second half of Mr. Barlow's volume, as we have already observed, is on subjects connected with midwifery. The papers, of which this part is composed, are partly new and partly reprinted from periodical Journals. Of the latter description are, 1, his Inquiry into the various Opinions advanced by Writers on Midwifery, respecting the Management of the Placenta; 2, on the Advantages and Disadvantages of inducing Premature Labour, with a view of superseding Embryulcia, the Section of the Symphysis Pubis, and the Cæsarean Operation; and, 3, Observations on Delivery in difficult Cases of the Presentation of the Shoulder of the Fetus, and where one or both Arms present along with the Head.

The public have long ago decided on the intrinsic value of the doctrines contained in these essays; nor are we disposed to disturb their verdict. It should, however, be observed, that considerable additions have been made by the author to each, and especially to the first, of the above-mentioned essays. We heartily agree with Mr. Barlow in the rules he has laid down for the removal of the placenta, in cases where it does not follow the delivery of the child in the space of from one to two hours; and also where a profuse hemorrhage takes place on the delivery of the child, and continues without restraint before the exit of the after-birth. There is no room for temporizing in either case; and the idea of leaving the management of the placenta, wholly, to nature, is perfectly absurd, not to say mischievous.

Under the head of distorted pelvis, we find the following synoptical table, exhibiting, at one view, every possible deviation betwixt the natural dimensions and the greatest degree of distortion.

| | *The distance from the upper edge of the Symphysis Pubis, to the superior part of the Os Sacrum, or conjugate diameter of the Pelvis.* | |
|---|---|---|
| Well-formed Pelvis. | From 5 to 4 inches. | Delivery by the efforts of nature alone. |
| I. First degree of deformed Pelvis. | From 4 to 3 or 2¾ inches. | Delivery by the efforts of nature, or assisted with the forceps or lever |
| II. Second degree. | From 2¾ to 2½ inches. | Premature Delivery. |
| III. Third degree. | From 2½ to 1½ inches. | Embryulcia, or Delivery with the Crotchet. |
| IV. Fourth degree. | From 2½ inch, to the lowest possible degree of distortion. | Cæsarean Operation. |

Mr. Barlow has again brought forward his successful case of Cæsarean operation, with which the medical profession are well acquainted. To it he has added two other cases, in which he performed the same operation, though not with equal success. The history of the latter case, though long, we must insert in this place, as an instructive lesson to practitioners in midwifery. We have no hesitation in asserting, that the delivery of the child can be effected, with the assistance of the perforator, where the anterio-posterior diameter is $1\frac{3}{4}$, as it appears to have been in Mr. Barlow's case. We have, on a former occasion, brought forward a case in point, where a diameter of $1\frac{5}{8}$ proved no impediment to the delivery of a large child, after cephalotomy had been performed.* At the same time, we do not mean to condemn Mr. Barlow for having had recourse to the Cæsarean section in his case, where he had a conviction of the child being alive; but he should have determined upon performing it sooner.

"The following case of Cæsarean operation occurred to Mr. Dugdale, surgeon, of Blackburn, by whom I was called in consultation; the history of which I will give in his own words.

"'Early on Friday morning, April 6th, 1821, I was desired to visit the wife of George Ridgedale, in the 42d year of her age, and in labour of her — child at the full period of gestation.

"'Her general health, I was informed by Mr. Barlow, who had attended her in several of her preceding labours, had suffered much for the last two or three years, during which period she had become much reduced in stature; and had not, for some months past, been able to walk without assistance, owing to a state of general debility of the system, induced by an anasarcous affection of both legs, and a state of malacosteon of the bones of the pelvis.

"'On my arrival, I was informed that she had had uterine pains upwards of twelve hours, which now recurred at regular periods every ten minutes.

"'On examination *per vaginam,* I found the os uteri dilated to near the extent of half-a-crown, the membranes protruding into the vagina; but could not feel the presenting part of the child.

"'At 6 o'clock P.M. her pains returned more frequently; the os uteri appeared, to the touch, somewhat more dilated than on my former visit, and the membranes so filled the vagina as rendered it hazardous to reach the brim of the pelvis without rupturing them.

"'At 9 o'clock P.M. the pains were unabated in frequency; but the membranes, on examination, were so flaccid during the interim of the action of the uterus, that I determined to rupture them, which was done by introducing my hand within the vagina; and I found the head of the child presenting at the brim of the pelvis, and resting on the edge of the pubis: it was moveable, and easily receded out of the reach by pressure with the point of the finger.

* See *Report of the Practice of Midwifery at the Westminster General Dispensary, for* 1818.

" 'The projection of the sacrum and lumbar vertebræ into the cavity of the pelvis, together with the pendulous state of the abdomen, rendered it wholly impossible for the head to enter the brim of that aperture.

" 'After rupturing the membranes, the pains returned every five minutes, and, on waiting an hour, I repeated my examination; but, finding the head not advanced, and considering her life in great danger, I thought of introducing the perforator, with the view of opening the head, and lessening its bulk by evacuating the brain. To this I was induced on reflecting that, in this species of diseased affection of the pelvis, the bones frequently yield, and allow the head to pass with greater freedom; which circumstance, I was since informed by Mr. Barlow, had occurred in two or three of her preceding labours. But, on passing the perforator up the vagina uteri, and on making the least pressure on the scalp with the point of the instrument, the head receded beyond the reach of the finger; and this project was, of course, abandoned.

" 'The extent of distortion of the pelvis, together with the debilitated state of the woman's system, convinced me that she would die in whatever way delivery was accomplished; I was, therefore, unwilling to destroy the child, particularly as she assured me that she had felt it move very lately.

" 'Thus situated, I judged it prudent to consult my friend and late preceptor, Mr. Barlow, before mentioned, and waited upon him for that purpose. Mr. Barlow readily yielded to my solicitation, and accompanied me to the patient's house about twelve o'clock the same evening, when we found her in a state of delirium. After waiting for some time, she became sensible and more composed.

" 'Mr. Barlow now made the necessary examination *per vaginam*, and remarked that the pelvis was more distorted by a state of malacosteon than on her preceding labour; on which occasion he had been compelled to deliver with the crotchet.

" 'On consulting together on the propriety and mode of delivery most proper to be adopted, it was our united opinion that the possibility of effecting this purpose with the crotchet was doubtful, and, if attempted, she might expire under the operation; and, as there was reason to believe the child to be alive, we concluded the Cæsarean section to be the most justifiable mode of proceeding.

" 'Our sentiments were immediately communicated to the woman and her friends, who expressed a wish to have the aid of Dr. Chew on the occasion. A messenger was immediately dispatched, and in a short time he arrived; and, having made a minute investigation of the state of the pelvis and condition of the woman, he coincided in our decision on the propriety of the operation, and she, without hesitation, submitted to our proposal.

" 'Mr. Barlow having attended her in most of her former labours, she expressed a wish that he would undertake the operation; to which he assented.

" 'Every thing necessary for the operation being prepared, Mr.

Barlow, about half-past three o'clock A.M., performed the operation in the following manner:

" 'The woman being laid on a bed in a horizontal position, with the head and shoulders a little raised by pillows, he commenced by making a longitudinal incision seven inches in length, beginning three inches above the umbilicus and two inches on the left side, carefully cutting through the muscles of the abdomen in a line parallel to the linea alba; by which the uterus came in view, and appeared in close contact with the abdominal parietes through the whole extent of the wound, from which a small quantity of serous fluid escaped.

" 'A corresponding incision was then made through the parietes of the uterus, which did not equal the edge of a shilling in thickness in any part, and to which the placenta firmly adhered throughout. In consequence of this attachment, it was thought prudent, rather than expose the woman to hemorrhage by detaching the placenta from the uterus, to continue the incision directly through its substance; which was soon effected, and the nates of the child exposed to view. The babe was then extracted alive, and, on the umbilical cord being tied and divided, was handed to a female assistant.

" 'The placenta and membrane were then brought away; and, during the contraction of the uterus, the bowels protruded at the wound, but were soon returned into their natural situation, and preserved there, while the incised integuments of the abdomen were closed together by means of the interrupted suture, about the space of an inch from each other, and secured with stripes of adhesive plaster, and pledgets of lint applied over, and a roller passed a few times round the body in the form of the T bandage.

" 'During the operation, and before the wound was closed, there was a quantity of blood discharged from the vessels of the uterus, which might probably amount to ten or twelve ounces.

" 'The woman never complained during the operation, nor seemed disposed to syncope. She was now removed, and laid in bed, and appeared much fatigued and sickly.

" '*April 7th, 7 o'clock* A.M.—Pulse 120 in the minute; pain of the abdomen; slight discharge per vaginam; with nausea and inclination to vomit.—℞. Tinct. Opij, gutt. xx.

" 'At 9 A.M. pains and nausea abated, pulse 120. Eleven o'clock A.M. pulse 130; tongue a little furred and dry; has very little pain, sickness, or retching.

" 'Not having made water since the operation, the catheter was introduced, and about a pint of urine drawn off. At 8 P.M. pulse 150; tongue moist; sickness and vomiting troublesome; does not complain of much pain; had no stool.

Catheteris introductio.

℞. Enema Cathart. statim infund.

℞. Tinct. Opij, gutt. xv. Sumantur post dejecerit alvus.

Eleven P.M. pulse 150; relieved by the Tinct. Opij, &c. Copious discharge *per vaginam*, and also from the wound.

Repetatur Enema Catharticum.

℞. Mistura Cathartica, ℥vj. capiat coch. duo magna omni hora donec alvus respondeat bis, et dein sumatur Tinc. Opij, gutt. xv.

" '*April 8th,* 8 *o'clock* A.M.—Pulse 130, and weaker; tongue furred, and dry with thirst. Abdomen tense and hard, with sickness and vomiting; profuse evacuation *per vaginam*; some discharge from the wound on removing the dressings. Had only one small stool.

Catheteris introductio.

At 12 o'clock merid. pulse 150; stomach rejects every thing she takes; other symptoms as before.

Repetatur Enema Cathart.

Half-past two o'clock P.M. pulse 150; no stool; vomiting continues.

℞. Hydrarg. Submuriat. gr. x; Opij. gr. j½. Mucilag. q. s. ut fiat pil. ij. sumatur statim.

" 'At 8 o'clock P.M. pulse 150; tongue furred and dry, with thirst and vomiting; abdomen tense. No stool.

Catheteris introductio.

Repetantur pilulæ catharticœ.

" '*April* 9.—Died about 8 o'clock A.M. fifty-two hours from the time of the operation.

" 'Eight hours after death, the abdomen appeared much distended. I obtained leave to evacuate its contents, with the intention of examining the state of the viscera and pelvis; but was prevented from making so correct an inspection as I could have wished, owing to the interruption of some of her friends.

" 'The incised edges of the integuments of the abdomen had not adhered in any part.

" 'On removing the sutures and enlarging the opening, a small quantity of limpid fluid passed off, similar to what was discharged during the operation.

" 'The stomach appeared much distended, from which near three quarts of dark-coloured liquid was evacuated.

" 'The liver was small and light-coloured.

" 'The intestines presented no appearance of inflammation.

" 'The uterus seemed free from inflammation, except a slight degree round the edge of the wound. The incision of this organ was contracted to about 2½ inches in extent; and its volume seemed of the usual bulk at the same period after delivery in the natural way.

" 'The dimensions of the pelvis were as follows:

" 'The conjugate or antero-posterior diameter, from the symphysis pubis to the nearest point of the sacrum or lumbar vertebra, 1¾ inch.

" 'The greatest space to be gained on the right side of the brim of the pelvis, 2 inches and near ¼; viz. from the anterior part of the acetabulum to the projecting part of the sacrum.

" 'The greatest distance to be obtained on the left side, between the corresponding points of the right aperture, 1¼ inch.

" 'The difference between the diameter of the two sides of the superior aperture, is manifested by the last lumbar vertebra projecting inwards, and forming a considerable curve in the cavity of the pelvis, induced by a state of malacosteon, which opposed the chief obstacle to embryulcia."

**Art. II.—*Observations on the Influence of Habits and Manners, National and Domestic, upon the Health and Organization of the Human Race; and particularly on the Effect of that Influence as it relates to the present State of English Females, in the higher and middle Classes of Life.* By Ralph Palin, m.d. 8vo. pp. x. 297. Hookham. London, 1822.**

Had Dr. Ralph Palin had any other christian-name than the one which appears on the title-page of his book, we should have proclaimed him either a native of the Gallic shores, or an inhabitant of a petty republic contiguous to the Jura. His ideas, not to mention his language, are exotic; and, if he be really an Englishman, then his residence on the continent has, in some respects, exerted a revolutionizing influence over his habits and manners. We only know of one other book in an English dress, which, for the importance of the title,—the obscurity of its language,—and the inutility of its conclusions, can be compared to the present volume; and that is Madame de Staël's Treatise on the Influence of the Passions, translated into English. We recollect, several years ago, rising from our desk, after having pored over her octavo for sundry hours, with a sensation of oppressive confusion in our head quite distressing, which, as it gradually vanished, on a due exposure to cool air, left a perfect blank on the tablets of our memory, as to what we had been reading.

Dr. Palin's book was laid on our table on the 22d of the month; and, although we do not profess to be friendly to disquisitions bordering on metaphysics, we were so caught by the title, that we soon got through its closely-printed pages; and this day, being the 23d instant, we sit down to give an impartial account of its contents.

It seems to be one of the objects of Dr. Palin to demonstrate a well-known truism, that the character and disposition of youth, whether in the moral or in the physical order, depend more on the early impressions received from parents, than on any subsequent ones which may be communicated by after circumstances. Dr. Palin adds this remark, that the female youth is more particularly liable to the above observation; so that the character of their constitution and temperament will be found to depend much more on the management of the early period of life, than of any more advanced one.

"By the early period here is meant that progressive stage, which precedes the full developing of the system; and, according to the principles which regulate the physical education during this progress, the constitution will become ameliorated, or otherwise, and the whole train of conservatory movements receive the impressions of strength or weakness." (Preface, p. v, vi.)

The sort of management, to which the author alludes, constitutes what foreign writers have called "Education physique;" a subject on which various works have been written abroad, though with very little benefit to mankind. In this country the subject of physical education has been wholly neglected; and, looking on Dr. Palin's work as a first attempt to introduce the consideration of that important subject to the notice of the English public, we hailed its appearance with satisfaction.

We shall let our author speak for himself, in giving a brief abstract of the matter contained in his volume.

"The two first chapters, which might form the first part, relate to the influence of climate upon the human system, connected with those impressions which attach to it from the action of the great phenomena of nature upon the peculiar phenomena of life, and with those modifications which it produces, under different circumstances, in the female constitution.

"The five subsequent chapters, which might be termed the second part, refer to the influence of artificial habits, as they are diversified by the effects of climate, upon the animal system, and to the modifications we have it in our power to make, according as the principles just mentioned shall direct our management of early life; for artificial manners and habits may have opposite effects, from the manner in which they are regulated: they may support the best views and designs of nature, or they may frustrate them.

"The eighth chapter, which might form the third part, refers to the consequences which, under our climate, often follow in the female constitution, when the principles, which ought to form the guide of their physical education, give place to others which produce opposite effects." (p. ix. x.)

With the subject of the first chapter, and its two sections, we, as medical men, can have but little to do. The first point treated in this book, on which we should arrest our attention, is the consideration of those physical causes which are supposed, by the author, to act in determining the character of the female sex. We do not think Dr. Palin at all happy in his positions on this head.

1. *Of the disposition which the female system acquires in the progress of its development.*—From the little activity, says Dr. Palin, given to the muscular and animal system, the nervous organ acquires in females greater mobility, and, by re-acting upon the former, renders it far more susceptible, and presents a form of superior sensibility and irritability. We acknowledge our inability to understand this passage. Females, "in our climate, and under our domestic institutions, are more arbitrarily submitted to the yoke of artificial laws, which communicate the impressions of debility to the organization, far more frequently than takes place in the other sex." This passage is not much clearer than the former. The corollary which seems intended to follow whatever doctrine it was the intention of Dr. Palin to incorporate in the above two passages, is contained in the annexed extract.

"It is this circumstance, and this peculiar susceptibility to receive the impressions of organic weakness, under our social habits, which renders the physical education of the female a subject of so much importance. Her constitutional disposition, partaking naturally more of activity than strength,—more of sensibility than firmness, becomes endued, under our institutions, with a peculiar irritability; which, being more active in early age than at any other period, makes the task of submitting her to their full influence one of great delicacy. In fact, in our subjection of this easily excitable frame to the laws of artificial life, we should be constantly guided by the indications of nature, as they appear manifested in the economy of the system. But attention to this subject becomes of greater importance, in proportion as the unfavourable effects of climate exercise a correspondent influence over the habits and manners of life, in producing an ungenial re-action of these upon the physical constitution. These effects, according to the preceding observations, will operate more in countries of a low temperature than the reverse. It seems, in fact, that, in contradiction to the common opinion, the stamina of females are more feeble in cold than in hot climates, as their muscular fibre is less firm and dense; and that the idea so usually entertained of the action of the former in strengthening the powers of life, is often erroneous, since the effects of that action are relative to habits. Cold, by the re-action it produces, and by the vigorous exercise it promotes, may be one source of strengthening the powers and developing the energies of the male, who lives in the open air and is engaged in active pursuits; but it has, usually, a very different influence upon females, when joined to the confinement and artificial temperature in which it obliges them to take refuge; and hence the influence of some climates upon the different sexes proves, in many respects, extremely opposite." (p. 36—38.)

2. *Of the causes which make climate and temperature act with a varied influence upon the different sexes.*—Dr. Palin is of opinion that the nature of the English climate is unfavourable to the female sex; for the confinement and seclusion which it renders necessary, has, on the female constitution, the most pernicious effects. To the absence of any such necessity in southern latitudes, must be attributed, principally, not only that firmness of fibre and physical energy, but those personal advantages which belong, in a superior degree, to the females of warm climates; for, with respect to beauty and expression of feature, and form of person, we must allow them to be greatly in favour of the inhabitants of the South.

"It were vain to look in the German nations, or amongst their northern neighbours, either for that firm texture of flesh, which is peculiar to the truly fine form, or for that expressive cast of feature or elegant mould of limb, which are common to the more southern females. The women of several parts of Germany, particularly the Saxons, display a fine complexion and beautiful bloom, but their light hair and fair skin are emblems of their debility; nor does the texture of the fibre prove greater strength. What a contrast do we find between the firmness of the flesh of the Italian and Spanish women, and the looseness and flaccidity of that of the Dutch, whose forms are almost uniformly weak and nervous, and have so languid a circulation, that artificial means are constantly employed to keep warm the feet! The same contrast exists, in a greater or less degree, between the female inhabitants of other climates, where there is equal diversity of temperature." (p. 42, 43.)

Our author indulges so often in speculations far above our comprehension, that we cannot attempt to follow him throughout his book. What seems to be the most important consideration respecting the general education of females, which Dr. Palin is desirous to impress on his readers in the two first chapters of his work, is that of the due regulation of the impressions arising from five causes:

"1st. The excitements which affect particular parts of the organic and muscular fabric under the influence of artificial habits;

"2dly. The stimulus of the animal system in exercise;

"3dly. The quantity of natural stimuli depending upon food, &c. &c.

"4thly. The stimuli of the cerebral functions and mental exertion in education; and

"5thly. The quantity of external stimuli depending upon clothing."

In chapter third, Dr. Palin proceeds to consider the effect of excitement, unequally applied to the muscular system in females. In speaking of the exciting causes of distortion of the spinal column, our author has added another to those generally enumerated by writers, which, though less considered, seems, amongst our females of the better classes, more active than the rest, and more frequently the source of deformity.

"This is the irregular action of the vertebral and extensor muscles, which support the body, from the fatiguing manner in which they are excited by the discipline of our domestic institutions. Such, at least, is the origin of the most common kind of deformity in females; which is caused by the highly imprudent way in which the observance of the erect position is enforced; and not only in frames where there exists a weakness of the physical organization, is this effect produced, from the cause just mentioned, but also in forms of greater strength." (p. 53.)

Having entered at large into the subject of the pernicious effects produced by certain excitements applied to the muscular system, on the outer form of females, Dr. Palin next considers the sort of unfavourable results which must arise out of the same causes, when acting on the internal organs. The state of extension in the muscular system is always that of great inconvenience, if long continued. Complete relaxation, in the recumbent position, for a proper time at mid-day, at least, should therefore, observes Dr. Palin, form a regular part of physical education in females under the influence of our domestic institutions.

We now pass on to the consideration of the effects which exercise and air have upon the muscular and animal system in females, at this early period of life, which Dr. Palin has dilated upon in the two sections of the fourth chapter. The females of the better classes in England suffer very considerably from a morbid susceptibility to atmospherical impressions, to which the confinement they suffer renders them peculiarly liable. Hence one of the most important points of their physical education, is that which relates to the strengthening of the constitutional energies, in increasing their power of resistance to those effects which arise from the unfavourable influence of the climate.

"We know that the most remarkable effect of habit, is to diminish gradually the sensibility of the organs; and that an habitual exposure to atmospherical vicissitudes produces this result in the most salutary degree. To this object, therefore, should the attention be particularly directed in the management of female life; for, in proportion as it is attained, the action of the functions will be more complete, and health and vigour be infused into the system." (p. 86.)

The same observation, but perhaps with greater force, may be made with regard to exercise.

"We observe that the fibre of the muscles acquires firmness and hardness in the degree that the animal takes exercise; and that, in proportion as the habit possesses firmness of fibre, it becomes capable of supporting exertions indicative of strength. From this circumstance, which shows the relation of organization to action, we may deduce the inferior adaptation of females for vigorous exercises, in comparison with the other sex; and the less necessity thereof to their health. In adult females, in whom we generally find the fleshy fibres feebler, and the cellular tissue more abundant and more loaded with fluids, than is the case in the male, much exercise, far from being necessary to preserve health, appears to exhaust the vital energies, and prematurely wear out the frame. This weakness of the muscles inspires a distaste for the more violent exertions, and impels, almost instinctively, to light amusements and sedentary occupations. The habitual sentiment of debility, which is partly the result of conformation, in diminishing the feeling of confidence, diverts from the employments that require it; and this predominant characteristic of the physical constitution seems to elicit certain moral qualities, and to produce that talent of finesse and address, which compensates, in a great measure, for the want of personal force. From this peculiarity of the physical and moral attributes arise those dispositions, tastes, and habits, which distinguish females from the other sex. The contexture of all their organs is more delicate, and their primitive constitutional dispositions are continually renewed, as it were, by the operations of their sensibility." (p. 86, 87.)

"The mode of exercise indicated in different subjects, whether of an active or passive kind, must, even in the period of youth, depend frequently on circumstances referrible to the constitution and habit; but, in a state of health, the former will generally be most congenial to the young. This will be necessarily, in many cases, an important consideration; because the effects of active and passive exercise, on the circulation,—on the secretions and exhalations,—on the digestive and respiratory organs, are very different. In fact, exercise, whether considered only as a source of amusement, or as a necessary part of the regimen of health, must, in order to answer either view, hold some relation to the ordinary habits and manners of life, as well as to age and constitution. An airing in a carriage, for example, though an excellent exercise for them whose minds and bodies have experienced the fatigue of previous occupation, and for the generality of adult females; will answer the preceding indications in a very inadequate manner in the case of the young, in whom all the muscles of volition are peculiarly disposed to action, from that excess of vitality natural to youth. It is then that the muscular and active exercises are more agreeable to the whole constitution of life, and that the expansion of the various powers and processes of vitality appears greatly to depend upon their employment." (p. 94, 95.)

The transition from the consideration of exercise and air on the development of the muscular system in females, to that of the effects of particular aliments on the animal fibre, is perfectly natural. To the latter, then, are we lead in succession by our author.

Regimen is not an unimportant part of the science of life; since, under its influence, we see organized bodies become so variously and so profoundly modified in their internal parts, as to lose those dispositions they had received from original conformation, and acquire a susceptibility to new impressions and new habits, which relate not only to the physical functions, but to the moral and intellectual ones. The consideration, therefore, of the subject of diet may be placed under two points of view; the first relating to the effects it produces on the muscular and organic systems; the second regarding the same effects produced on the nerves.

Dr. Palin lays down some general, as well as specific, rules with regard to the admission of a certain quantity of stimulus (food) into the stomach.

In speaking of vegetable diet, our author has advanced some ingenious arguments against its supporters, whom he supposes to recommend vegetable, in lieu of animal food, as containing less stimulating matter, and furnishing the principal part of the nutriment employed in a state of nature.

"But it may be observed to them who argue on natural instincts, that the vegetable food of cultivation is very different from that of nature. In countries where a vegetable diet is the principal source of support, the constituent principles originally imparted to it by nature are less acted upon, and less changed, by the intrusive arts of cultivation, than in climates like our own, where all the product of horticulture is the fruit of labour. While, by cultivation, we increase and improve, in the highest degree, some of the properties of vegetables, we diminish and destroy others, which are of the most essential use in the process of digestion. There is naturally a bitter principle in vegetable bodies, which is as necessary to the assimilating process of herbivorous animals, as salt is to that of the carnivorous classes. By cultivation we seem to decrease the bitterness, in proportion as we increase the nutritive qualities of vegetable substances. It has been assumed, indeed, that the importance of the former is in an inverse ratio with that of the last. But the effects of some of our nutritious vegetables upon the herbivorous race, seem to show that there are some exceptions to this conclusion. Mr. Sinclair, in his researches, which are recorded in the "Hortus Gramineus Wobernensis," remarks that, if sheep are fed on yellow turnips, which contain little or no extractive, they are led instinctively to seek for provender which may contain it; which if they cannot find, they become diseased and die. The same person has demonstrated that no cattle will thrive upon grass whose constituent parts do not partake, in a certain degree, of the bitter principle, which acts as a natural stimulant, and forms an essential ingredient in the provender of herbivorous animals.

"Moreover, climate is another cause which produces powerful impressions and remarkable changes, equally on vegetable as on animal life. How cogently this cause operates on the former, we have the most marked evidence in its influence upon those plants, whose effects on the animal frame are most striking and powerful. The plant which, under the sun of one country, and while flourishing in its *solum natale*, is uniformly narcotic, may become, by removal into another soil, cathartic, or assume some other equally opposite constituent property from change of place; the qualities which particularly distinguished it in one situation, being greatly diminished or lost in another. Thus senna, by transplantation from Arabia into the south of France, undergoes as great a change in its virtues as in its physiognomy. Correspondent changes take place in other instances; and, from analogy, we must conclude that similar effects of climate are experienced, in a greater or less degree, by the whole vegetable class, under changes of climate and of cultivation, both of which alike vary the relative qualities of the constituent principles of plants. Since then, under the operation of these powerful agents, we see the whole order of vegetable life put on such complete metamorphoses, and change their elemental properties and virtues with the same facility that they change their external aspect, the argument, so often employed in favour of a vegetable diet, that it is a return to nature, can by no means apply under the artificial race of vegetables which horticulture has created in our northernly climate." (p. 127—129.)

The question respecting diet, as far as concerns our species, seems not to be what particular aliment the natural conformation of our organs qualifies us originally to take, but what best suits the different circumstances of life, under the great and peculiar changes it undergoes in the various modifications it assumes. Hence, though the question as to what was the particular food destined by nature for mankind, may be a point for agreeable or profound speculation, it can scarcely be deemed one of such great practical utility as some would persuade us; and perhaps, impartially pursued, it would bring us to the conclusion of DAUBENTON, that man has no natural food.

"In the physical education of females, the choice of diet must have some relation to those diseases to which the delicate state of their organs render them most susceptible. Climate not only modifies the properties of many articles of aliment, by the action it produces in their constituent parts, but varies the degree of adaptation on the part of the organs to their impression. But the influence of climate, which it behoves us most to be observant of, is the general determination it sometimes manifests in infusing the propensity to particular trains of diseases; and the effects of regimen, which it is equally most important to study, are those which operate to counteract similar propensities. Now, the prominent morbid action of the atmospherical phenomena we experience, seems to be that which disposes, almost uniformly, to the diseases of debility; in correcting the predisposition to which, a proper regimen, employed sufficiently early, may have great influence as a preventative. Such constitutional debility is the primary source of every species of scrofulous affection; whether connected with a morbid irritability, exciting particular parts, or with a general torpor extending through the whole frame, and connected with a deranged and altered state of the glandular organs. The same constitutional delicacy disposes to pulmonary consumption, in endowing the internal membrane of the lungs with a too-great susceptibility to certain agents, on the operation of which depends the development of disease. This debilitated condition of the living fibre, leading to a diseased action, is totally independent, however, of any acrimonious impregnation either of solids or fluids; and therefore in females, whose early tendencies lead to well-grounded apprehensions of some future visitation from complaints of a similar character, preventive measures cannot begin too soon. Their success indeed will depend, in great measure, on that part of physical education which relates to the dietetic plan, and on the proper adaptation of regimen to early indications and latent dispositions." (p. 136—138.)

Dr. Palin examines three different temperaments which females are more apt to exhibit, and to each he appropriates a particular sort of diet and a specific regimen. This subdivision is, to say the most of it, ingenious. In practice, all these distinctions vanish; and we often find that a female, possessing a temperament marked by decreased irritability, cannot well be managed without a diet which Dr. Palin has assigned to females of a highly nervous disposition. As for his observations on the effect of certain diet on the nervous system and the morals and happiness of the social order, they are, by far, too metaphysical to be noticed in a medical review. Indeed, we fear that our readers are already disposed to find fault with us for dilating at such length on a performance, from which we have not been able to cull any very useful practical precepts, applicable to the purposes of life, that had not already been very often proclaimed by preceding writers. We must, therefore, hasten on to the conclusion, simply enumerating the remaining subjects, on which Dr. Palin's pen has touched with equal felicity.

The first of these is the sympathy between the animal and intellectual system, and the influence of the action of the former in allaying the irritability of the last.

Next we have some observations on the manner in which, from the sympathy between the muscular and intellectual system, mental excitement may act in allaying corporeal irritability.

In chapter the seventh, the author has undertaken to examine the very important question of clothing, and its relation to health, under different circumstances connected with atmospherical impressions. With regard to the female sex in particular, this question is one of great interest. Pectoral congestions, in climates like our own, are met with more frequently than in colder climates, from the chest being oftener left exposed to the inclemency of the weather, and from the inadequate attention paid to the external coverings of the body.

"In what manner the negligence with respect to the coverings of the body may act in producing thoracic inflammation, or other fatal affections of that organ, at the period of life when they most frequently, may be explained in the words of a physician of the first character and celebrity.—" The branches of the intercostal vessels, which are very numerous, piercing through the substance of the intercostal muscles, communicate a good deal by anastomosis with the external vessels on the sides of the chest. Hence, whatever may act upon these external vessels, so as to excite contraction in them, may be supposed capable of producing an accumulation of blood, as well as an increased action in the inner branches of the intercostals, many of which are distributed upon the pleura. Many of the inhabitants of this country, from their mode of dress, have their chests much exposed to the influence of a cold and very uncertain climate, and hence the blood is frequently thrown inwards into the small vessels ramifying upon the pleura. For these reasons, probably, the pleura is more liable to inflammation than other membranes investing cavities which have no external opening. This is so much the case, that one can hardly examine the chest of any person who has arrived at the adult state, without perceiving more or less the traces of a present or former inflammation." (p. 226—227.)

There can be no doubt that diseases connected with consumption, the origin of which is congenital, may remain latent through a long life, or be brought early into fatal activity, by a greater or lesser degree of that attention which regulates the external covering of the body, and establishes the healthy condition of the skin.

To regulate, therefore, the effects of atmospherical impression on the female constitution, Dr. Palin proposes, 1st, that we should direct our efforts towards lessening, as far as possible, the susceptibility of the habit to atmospherical impressions; and, 2dly, that we should guard against them by proper coverings.

The terminating chapter of Dr. Palin's work involves matters of practical importance, which he has treated in a very able manner, and to which we must refer our readers, certain that they shall find what will repay them for their pains. We more particularly allude to his observations on the scrofulous diathesis and temperament, and on the consumptive tendency in early life.

In characterizing the performance before us, we should say, that the language of it is frequently obscure, and that the author's meaning is not always clearly defined. There are innumerable repetitions in the body of the work, and an ever-recurring strain of metaphysical ratiocination, which we should not have expected in a modern book on the physical education of females; for, whatever Dr. Ralph Palin may say, or the admirers of his system repeat, the wearing very tight stays, and a long waist, may injure the digestive organs of a young female, and make her an old invalid, but will never prevent her becoming a STAEL or a GENLIS.

---

ART. III.—*Further Observations on Strictures of the Rectum, with Remarks on the Opinions of some late Writers relative to the Situation of the Disease; and also on Spasmodic Constriction of the Sphincter Ani, with a Translation of Part of* M. BOYER'S *valuable Paper on that Complaint.* By W. WHITE, Member of the Royal College of Surgeons, London, &c. 8vo. pp. 105. Bath. 1822.

IN every branch of science nothing is so useful as the diversity of opinion, which we frequently find existing among men who have investigated the same subject. It produces doubt in the minds of those who are anxious to attain knowledge; and leads them to receive, with caution, doctrines to which they would otherwise implicitly subscribe, until by their personal enquiries they are convinced of their orthodoxy. This remark applies to Surgery, as well as to every other department of art and science: and it is the anticipation of the beneficial result of such doubt, as far as regards the subject of the treatise before us, that induces us to lay an analysis of the opinions it supports before our readers.

In his preface, Mr. WHITE states, that the present publication has been drawn up for the praise-worthy purpose of preventing young practitioners from being misled by "some representations" contained in the writings of Mr. CHARLES BELL and Mr. HOWSHIP on Strictures of the Rectum, calculated to impress "the minds of the inexperienced with a limited and partial view of the complaint; which, if adopted, would tend to prevent the knowledge of it from extending." Mr. White complains, also, that these authors, although their publications contain "an evident allusion" to some of his remarks, yet have not mentioned his name; an oversight, however, which, we must remark, is by no means peculiar to the accused, nor at all uncommon in the present period.

Mr. White's leading principles are, that stricture of the rectum is not, as has been maintained by other writers, always the effect of inflammation; and that the inner membrane of the rectum is not the seat of the complaint. His opinion, with regard to the absence of inflammation, he states, to be founded on the following observations:—

"The great length of time the disease is frequently known to exist—the slowness of its progress—its limited nature, only occupying a very small portion in the circumference of the intestine—and the inner membrane having been frequently found in a healthy state on dissection."

The correctness of our author's conclusions from these premises we shall afterwards examine.

The majority of authors have taught that the disease commonly occurs near the extremity of the gut, or at least within the reach of the finger; but they have not certainly maintained, as our author would imply, that it is not also sometimes situated higher up in the gut. He is more successful in refuting the hasty opinion of Mr. SHAW, that the sacrum offers such an obstacle to the introduction of the bougie "further than six inches into the rectum," as to occasion the error of supposing, that the difficulty is caused by a stricture of the gut.

"If the projection of the sacrum," says our author, "be always mistaken for stricture, according to Mr. Shaw's conjecture, I should be glad to know, why so many persons have been completely relieved, from the most distressing symptoms, by the use of the bougie, when all other means had failed, if no real obstruction had existed in the intestine?"

And to prove that his objections to the commonly received opinion regarding the seat of the disease are not hypothetical, he adds—

"Although I may venture to state, that I have as frequently met with a contracted state of the rectum, towards its lower extremity, under as many different forms as any other practitioner; at the same time it may be observed, had my knowledge of simple stricture depended on those cases merely which have occurred at the lower extremity of the gut, it would have been extremely limited indeed; and, in all probability, I should not have seen any reason for deviating from the commonly received opinion. But, it has so happened, that in the course of an extensive practice, very few cases of the simple form of constriction have occurred so low down the rectum as to be within reach of the finger. And I can positively assert, that the disease has been frequently overlooked, when the rectum had been subjected to an examination by the finger only.

"So seldom does simple stricture take place within reach of the finger, that on looking over a list containing one hundred and eighteen cases, I do not recollect meeting with half a dozen, out of that number, that were within reach; and even these, in all probability, would not have been discovered by that mode of examination, had not the intestine been distended in consequence of a lodgment of fæces immediately above the stricture, or passing through its orifice at the time; by which means the stricture was brought within reach; and an opportunity was likewise afforded of ascertaining the nature of the constriction."

Returning to the consideration of stricture of the rectum, the cause which he has, already, declared is not inflammation, Mr. White gives the following as his theory of the origin of the disease:—

"Any cause, therefore, which tends to obstruct the passage of the fæces through the canal, must necessarily excite the fibres of the muscular coat of the intestine to a greater action than ordinary, for the purpose of expelling the fæces; this inordinate action, frequently excited at the narrow part of the canal, will, in all probability, sooner or later, terminate in a permanent spasmodic state of the muscular fibres of that part. How long a permanent contraction of the muscular fibres may continue, before any alteration in the structure of the part takes place, it is impossible to conjecture: perhaps under very favourable circumstances, the disease may not proceed to any other structural derangement: but, there is too much reason to apprehend, from various cases of dissection, that, in general, disorganization, sooner or later, takes place; which consists in a thickening of the coats of the intestine, particularly its muscular coat, probably from a very gradual deposition of coagulable lymph between its fibres."

Now we must confess that we do not exactly comprehend the force of our author's reasoning. We cannot conceive the possibility of any degree of spasmodic contraction of the muscular part taking place, sufficient to occasion a permanent contraction of that part, without the existence of inflammatory action. The very occurrence of spasm implies the presence of an irritating cause, the operation of which on the irritability of the part produces contraction of the muscular fibres; but this contraction must be necessarily followed by relaxation, whether the cause which induced the contraction be withdrawn or continued to be applied; for in the first instance the excitement being sufficient only to produce a limited effect, the state it induces must cease, when the impulse which the exciting cause impressed is exhausted; and in the second the effect of the application of any inordinate stimulus, to an irritable surface, cannot be maintained by the continued presence of the exciting cause; for the part loses its susceptibility by the habit induced from the continued application of the same stimulus. Inflammation, however, may be produced by powerful and long continued spasmodic contraction in the rectum; and it is easy to conceive that the coagulable lymph, thrown out between the coats of the intestine, will produce such a diminished diameter of the part as would not only prevent the fæces from passing readily through it; but, by the retention of a portion of them, will occasion a preternatural extention of the gut above the stricture; and thus by an increase of the morbid excitement the stricture will not only become more formidable, but ulceration may follow. Indeed, in every case of stricture, whether of the œsophagus, the rectum, or the urethra, we cannot conceive the existence of a thickening of the lining membrane of the part, and the contraction of the passage consequent upon that thickening, independently of inflammation. So far we are of opinion that our author has not made good his point, on this part of his subject.

Mr. White next combats the opinion of Mr. Howship that the contraction of the sphincter ani, which is sometimes accompanied by fissure, is the consequence of a venereal taint: and he observes that the contraction "which is sometimes found at the anus as a consequence of venereal infection, differs materially from spasmodic contraction, in being always attended with more or less structural derangement." We admit the distinct character of the two diseases, but not from the circumstance which our author points out as the characteristic feature of the venereal constriction; for we cannot form any idea of fissure of the sphincter existing without the presence of structural derangement. Our author expresses much surprise that neither Mr. Charles Bell nor Mr. Howship have noticed Mr. Boyer's observations on this form of the complaint; and, to make up for the deficiency of these writers, he has translated a very considerable portion of the memoir of that celebrated surgeon. For the benefit of such of our readers as may not have an opportunity of seeing either the original* or Mr. White's translation, we quote that part of the translation which describes the method of operating which Mr. Boyer has successfully employed for the cure of constriction of the sphincter, with or without fissure.

"Now for the manner in which I perform the operation—the patient takes three days before a mild purgative, and the same day a laxative enema to evacuate the intestinal canal, in order that the patient may remain some days without being affected by a desire to go to stool.

"I make him lie upon his side, as for the operation for fistula in ano; I carry the fore-finger of my left-hand, anointed with cerate, into the rectum, and upon my finger I make a bistoury glide on its flat side, the blade of which is very narrow, square at the end, and the extremity rounded off. The edge of the bistoury is then directed towards the right or left side, according to the place which the fissure occupies, and with one incision I divide the intestinal membranes, the sphincters, the cellular tissue, and the integuments of the nates. I thus form a triangular wound, the top of which reaches to the intestine, and the base to the skin; it is sometimes necessary to elongate this, I do this with a second cut of the bistoury. In some cases the intestine slips away from the edge of the instrument, and the wound of the cellular tissue extends higher than that of the intestine; we must then introduce the bistoury a second time into the rectum to lengthen the incision of the intestine, or complete it with the blunt pointed scissars.

"When the constriction is great, I make two similar incisions, one to the right and the other to the left; and when the fissure is situated before or behind, I do not comprehend it in the incision.

"We introduce immediately into the wound, or the two wounds, a large bougie, which prevents the edges of the incised parts from re-uniting in an irregular manner. We plug it up slightly with lint, apply a number of pretty long compresses, and the whole is supported by a bandage, like that which is used for fistula in ano. It is seldom that hæmorrhage supervenes, a slight compression is always sufficient to stop it. We do not remove the first dressing for three or four days, and afterwards dress it every day till the cicatrix is entirely formed; this is generally a month or six weeks, in some circumstances the cicatrization has not taken place till after the second month, or in the course of the third; but at other times, also, in twenty days—once only in fifteen.

"All the patients on whom I have performed this operation, have been cured radically, completely, and without return of the pain of the fissure, or the constriction."

* The original will be found in the *Journal Complementaire du Dictionaire des Sciences Medicales* for November 1818.

Our author considers the constriction of the sphincter to be, almost in every instance, connected with stricture of the rectum, and, consequently, a secondary affection: and adds, that previous to the publication of the former edition of his observations, he had not "met with any case that did not yield to the use of the bougie; but two cases have occurred since, where there was a necessity for dividing the sphincter: though," he adds, "I believe this operation will be found very seldom necessary when the bougie is judiciously managed." p. 34. Our author, however, admits the importance of the operation introduced by M. Boyer; and we certainly consider it one of the greatest improvements, in the treatment of this variety of stricture, which has been proposed.

Mr. White concludes his remarks by a cursory view of the influence of piles, excrescences, and prolapsus ani, in producing stricture of the rectum; and points out the advantages to be derived from the use of the bougie in hæmorrhoidal tumors, when inflammation is not present or has been previously subdued. He has lately employed Mr. Arnot's dilator, and delivers the following opinion regarding its applicability as a distending agent in contractions of the rectum.

"It is a very neat and ingenious contrivance. One great advantage attending its introduction is the distending power not being applied until the instrument has passed throngh the stricture. It was a long time before I could get one made completely air tight, and even then, I am inclined to think there is an uncertainty of its remaining all the while sufficiently distended at the stricture, as to fully answer the purpose. Patients in general complain that withdrawing it produces more pain than the bougie, so that they do not like it so well. Although it may not supersede the use of the bougie, yet I think its occasional use may be attended with advantage.

"I would just observe, that being unable to introduce the dilater (as contrived by Mr. Arnot) in consequence of the metallic tube not yielding to the curvature of the passage where the stricture is high up, I had the dilater fixed on a smaller elastic gum catheter instead of the metallic tube, which passes with great ease.

"This information will be found useful to those practitioners who may be disposed to make trial of the dilater in strictures of the rectum."

Sixteen cases, illustrative of the author's view of the disease in question, are detailed at length. As we cannot abridge these with benefit to our readers, we shall mention the heads merely of the majority of them, with any uncommon fact or peculiarity of practice; and extract entire one or two of those which are the most interesting. The first case is one of *Stricture with Rupture of the Colon:* in which the only circumstance worthy of remark is, that the vomiting and sickness, which continued for two days, "entirely ceased in consequence of the patient having been directed to take some curds and whey; the good effects of which," adds Mr. White, "I had often experienced in cases of obstinate vomiting." The next four cases, which did not come under the care of our author, are instances of distention of the colon, followed by rupture of the coats of the intestine. The sixth case is one of *Stricture with Derangement of the Colon;* which was cured by the use of the bougie conjointly with a course of aperient medicines. In these cases our author justly considers the distention of the colon to have been the consequence of stricture of the rectum.

The seventh and eighth cases are instances of *Stricture with Spasmodic Constriction of the Sphincter Ani.* We shall extract the first of these, as it is illustrative of the beneficial effects of the operation proposed and first performed by M. Boyer, for the relief of this variety of stricture.

"M. Tidcomb, aged forty, had been ill about two years. She complained of having been frequently troubled with a pain about the pit of the stomach, accompanied by a great sense of heat. She was often annoyed with distention of the bowels from wind, and experienced great difficulty in passing it downward. She was naturally of a costive habit of body, and commonly went three or four days without having an evacuation, and not then unless she took an aperient; but even with that assistance the motions were never satisfactory, and always attended with considerable pain; which continued several hours afterwards, at the extremity of the rectum. She had not passed any solid stools for a great length of time, and when she last observed them to be figured, they were very small and flat. Menstruation was regular but always painful. Her appetite was tolerably good, though sometimes she had sickness. She had been under the care of different medical gentlemen without deriving any advantage; but, on consulting Dr. Barlow, he suspected some disease of the rectum, and requested an examination might be made. On attempting to introduce the finger, the resistance to its passing was very considerable, from the strong action of the sphincter ani—it was accompanied by a fissure in a line with the os coccygis. There was also a stricture a few inches higher up the rectum.

"The patient was directed to take castor-oil every morning—to use a hip-bath daily: and an injection with a few grains of extr. papaveris—a bougie was also employed for some time, but the spasmodic action of the sphincter was so extremely distressing, as to render her incapable of persevering in its use. In consequence of which, recourse was had to dividing the sphicter by the bistoury, in the same manner as directed by M. Boyer. The sphincter was divided on both sides—a day or two afterwards, the evacuations were far less painful in passing, than they had been previously to the operation; notwithstanding the soreness of the part. Short tents made of lint covered with soft ointment, were employed, until the passages was able to bear the introduction of a bougie. The spasms at the sphincter entirely ceased, and the evacuations were discharged without pain or difficulty. In short, the great relief derived from the operation, would have led me to conclude, that a perfect cure had been effected, if I had not previously known a stricture existed higher up; which for some time required the use of the bougie. She was then able to undertake a servant's place, and some time afterwards she called upon me; and expressed great gratitude for the benefit she had received."

Next follow three cases of *Stricture with Irritability of the Stomach;* the most interesting of which is written by the patient, a lady. Case xii. is, also, drawn up by the patient, a lady, and details the success of the employment of the bougie in *Stricture attended with Hæmorrhoidal Tumors and Prolapsus Ani;* and the fifteenth case relates an instance of *Stricture with morbid irritability of the Bladder*, which, as the statement of the symptoms is written by the patient himself, and one of the strictures was between seven and eight inches up the rectum, we shall extract entire; and, with it, finish our remarks upon this little treatise; which has the merit of illustrating the fact, that much is yet to be learned regarding strictures of the rectum, and directing the attention of the profession to the further investigation of the subject.

"A gentleman between thirty and forty years of age, on his application to me, presented the following statement:—'About two years and a half since, I went to the late Mr. —— of Clifton, who sounded the bladder, as the symptoms I then felt led to a suspicion of my labouring under that complaint: but he pronounced the bladder to be free from stone, and the urethra from stricture: what was prescribed afforded me little relief. On returning from London the latter end of last May, I was dreadfully ill, having a great deal of fever, and violent inflammation. A dozen leeches were applied to the perineum, but I was no better. There was a sense of fulness in the bowels, which were evidently swollen; and sensibly aggravated after taking food—uneasiness in the rectum on going to stool, attended with great difficulty in voiding the fæces, which were generally discharged with a squirt; and after the evacuation a sensation, as though not half the fæces had been expelled. Perhaps repeated fruitless efforts to pass a stool eight or ten times a-day, with a manifest sense of constriction and tenesmus high up the rectum. Extreme suffering about the bladder and prostate gland, with ten or a dozen calls to make water during the night; and several attempts without succeeding, especially when wanting to go to stool, which was as distressing as the act of urining. The quantity voided was very small, the first portion tolerably clear, then it came off, having a turbid appearance, resembling chalk and water, followed by a dark liquid, the colour of coffee. The last-mentioned symptoms were much relieved by Dr. W——'s prescription of uvæ ursi, &c. Of the different opening medicines I took, castor-oil was the most efficacious and soothing—and, notwithstanding three or four enemas were thrown up in the course of the day, still there was a difficulty in procuring an evacuation. I do not recollect having had a natural or figured stool, such as confirms the bowels to be in a healthy state, for years past.

'Having been alarmingly ill five weeks since, from my bowels becoming very much swollen, with constipation, and the abdomen sore to pressure of the hand; it was suggested to try the use of a small candle, thirty-six to the pound: and after repeated efforts the small end passed through a stricture, causing it to bleed—then a small urethra bougie was used, the evacuation afterwards was in quantity astonishing. After this, I introduced a rectum bougie, which was extremely painful, and long before it could pass; and then with a jerk or slip, like going through a horny substance, it caused the right testis to swell immediately, and become very painful, with sickness of the stomach, so that the day after using the bougie I was very ill, and felt exceedingly sore high up the rectum, particularly after a stool of any consistency. I think the common bougie is productive of much irritation*.'

"As soon as the gentleman was able, he came to Bath; and on examining the rectum, I found two considerable strictures, attended with great morbid irritability of the intestine, which I have no doubt had been occasioned by the injudicious use of the bougies he had employed. The first stricture was about four inches up the rectum, and the second between seven and eight inches; the patient was sensible of the existence of two strictures, from his having passed the bougie beyond the second, although he had not noticed that circumstance in his statement. I began using a small-sized bougie, and the facility with which it passed the strictures, convinced him that he must have injured the passage, by using too great violence with the common bougie. He continued to take a little castor-oil every night, and to throw up an injection of warm water and sweet-oil once or twice a-day. He was also directed to take a few grains of extr. papaveris every night at bed-time. The bougie was gradually enlarged. In a short time, the gentleman returned home in a comfortable state, having been properly instructed in the use of the bougie. He, however, occasionally visited Bath, when I had the satisfaction each time to find progressive improvement. Unfortunately, soon after, imagining himself to be quite well, he omitted using the bougie regularly. At the same time he made too free in eating and drinking, with too much horse exercise, which brought on great pain of the bowels, with distention and obstinate constipation; that there was much difficulty to overcome the obstruction. He came to me soon afterwards, when he appeared conscious of the impropriety of his conduct, and sensible of the imminent danger into which he had brought himself thereby. There was great tenderness and irritability of the rectum, but with care and attention he soon recovered his former comfortable state, and returned home.

"It should be observed, the patient particularly noticed, that in proportion as the strictures had given way, the irritability of the

* "Practitioners are highly culpable, in suffering patients in the first instance, to use the bougie themselves, where so much judgment is requisite for its successful employment. I have known several instances where the bougie had been recommended without any examination whatever of the rectum!"

bladder lessened, and the water became clear. And although his bowels still required the assistance of medicine, he had entirely lost that frequent ineffectual desire to go to the night-chair, and tenesmus, with which he had been so much annoyed."

